# Pathogens detected in the tick *Haemaphysalis concinna* in Western Poland: known and unknown threats

**DOI:** 10.1007/s10493-021-00647-x

**Published:** 2021-08-11

**Authors:** Dorota Dwużnik-Szarek, Ewa Julia Mierzejewska, Mohammed Alsarraf, Mustafa Alsarraf, Anna Bajer

**Affiliations:** Department of Eco-Epidemiology of Parasitic Diseases, Institute of Developmental Biology and Biomedical Sciences, Faculty of Biology, University of Warsaw, Miecznikowa 1, 02-096 Warsaw, Poland

**Keywords:** *Haemaphysalis concinna*, *Babesia* sp., *Borrelia afzelii*, *Rickettsia* sp., Western Poland, *Alexandromys oeconomus*

## Abstract

In recent years, a new focus of the relict tick *Haemaphysalis concinna* was discovered in Western Poland, near Wolsztyn, Greater Poland voivodeship. This species may play an important role in the circulation of pathogens of medical and veterinary importance. In the present study we tested 880 juvenile ticks collected from rodents, including 427 *H. concinna*, 443 *Ixodes ricinus* and 10 *Dermacentor reticulatus* for three of the most common pathogens vectored by ticks in Poland: *Rickettsia* and *Babesia* spp. and *Borrelia burgdorferi* s.l. Additionally, molecular techniques were applied for accurate identification of tick host species (the voles *Microtus* and *Alexandromys*). Our study found differences in the range and prevalence of vectored pathogens between the three tick species. DNA of all three pathogens was found in *I. ricinus*. In juvenile *H. concinna*, DNA of *Babesia microti*, *Borrelia afzelii* and *Rickettsia* sp. was identified. Moreover, DNA of a new unnamed *Babesia* species related to *B. crassa*, was found in two *H. concinna* nymphs. This genotype of *Babesia* was previously identified in *H. concinna* in the Far East and then in Central Europe. DNA of *Rickettsia raoulti* and *B. afzelii* was detected in *D. reticulatus* nymphs*.* Among rodent hosts, *Alexandromys oeconomus* seems to be host of the highest significance for juvenile tick stages and was the only host species with *B. afzelii* detected in blood samples. Using phylogenetic methods, we confirmed a clear division between rodents from the genera *Microtus* and *Alexandromys*. Moreover, we found that *A. oeconomus* trapped in Western Poland clustered with a Central European *A. oeconomus* allopatric phylogroup.

**Supplementary Information:**

The online version contains supplementary material available at 10.1007/s10493-021-00647-x.

## Introduction

The relict tick *Haemaphysalis concinna* occurs in Europe and Asia in isolated limited locations (Rubel et al. 2018). Together with *Ixodes ricinus* and *Dermacentor reticulatus*, *H. concinna* constitutes an important element of the ectoparasite community of domestic or free-living animals and humans (Duscher et al. 2013; Rubel et al. 2018). Adult ticks infest wildlife and farm animals (cattle, goats and sheep), larvae and nymphs feed on small rodents, birds or reptiles. Humans can be attacked by both nymphs and adult ticks (Rubel et al. 2018).

In Poland until 2018, only a single female of this species was found on a cow near Troszyn, in close proximity to the German-Polish border and the Baltic Sea shore, in West Pomerania in 1953 (Lachmajer et al. 1956). In 2018 several new foci of *H. concinna* were discovered in Western Poland (Dwużnik et al 2019a; Kiewra et al. 2019). In total, 43 *H. concinna* specimens were collected in six out of 24 monitored sites in Lower Silesia (dolnośląskie voivodeship) (Kiewra et al. 2019). Additionally, in summer 2018 all stages of *H. concinna* were collected from rodents and vegetation near Wolsztyn, in Greater Poland voivodeship (Dwużnik et al. 2019a).

Knowledge on pathogens vectored by *H. concinna* is still quite limited, likely due to the difficulties in collecting ticks, because of the limited geographical range and low tick density (Rubel et al. 2018). This tick species may act as a vector of the *Rickettsia* bacteria, etiological agents of TIBOLA/DEBONEL (tick-borne lymphadenopathy/*Dermacentor* spp.-borne necrosis-erythema-lymphadenopathy) (Rieg et al. 2011). Moreover, *Borrelia burgdorferi* s.l. was also detected in *H. concinna* (Rigo et al. 2011).

In recent years, we have completed a large project on the vector role of juvenile *D. reticulatus* obtained from rodents (Dwużnik et al. 2019a, b). Rodents play a pivotal role as the hosts for juvenile ticks, larvae and nymphs, of at least three important tick species (*H. concinna*, *D. reticulatus* and *I. ricinus*) (Dwużnik et al. 2019a, b). In the present study we assessed the possible role of *H. concinna* ticks as vectors of pathogens from the genera *Rickettsia*, *Borrelia* and *Babesia*. Because a tick species can carry multiple pathogens, we examined the vector specificity of three tick species collected from rodents (*H. concinna*, *I. ricinus* and *D. reticulatus*). Co-occurrence of pathogens was also investigated as co-feeding of different tick species on one host can contribute to cross-species pathogen transmission (Dwużnik et al. 2019b). Finally, we compared the detection of pathogen DNA in both juvenile ticks (*H. concinna*, *I. ricinus*, *D. reticulatus*) and their host individuals, to verify to which extent positive results obtained in molecular testing of ticks reflect infection of hosts, the source of tick blood meal (phenomenon of ‘meal contamination’) (Dwużnik et al. 2019b).

## Materials and methods

All procedures involving rodent trapping and tick collection were described in detail in Dwużnik et al. (2019a, b). In total, 880 ticks belonging to three species were collected from 39 rodent hosts: 10 nymphs of *D. reticulatus*, 443 juvenile *I. ricinus* (405 larvae and 38 nymphs) and 427 juvenile *H. concinna* (405 larvae and 22 nymphs) (Table 1). Ticks were identified by a key (Estrada-Peńa et al. 2004) and by molecular methods (Dwużnik et al. 2019a, b). Then, ticks were tested for the presence of DNA of three pathogens (*Rickettsia* spp., *Babesia* spp. and *Borrelia burgdorferi* s.l*.*).

**Table 1:** Number of tick samples (larvae and nymphs) collected from various rodents

Rodent hosts	*Haemaphysalis concinna*			*Ixodes ricinus*			*Dermacentor reticulatus*
	Larvae (pools)	Nymphs	Total	Larvae (pools)	Nymphs	Total	Nymphs
*Alexandromys oeconomus* (n = 28)	393 (46)	22	68	318 (43)	36	79	10
*Microtus agrestis* (n = 2)	11 (2)	0	2	18 (2)	0	2	0
Total *Microtus* + *Alexandromys* (n = 30)	404 (48)	22	70	336 (45)	36	81	10
*Apodemus agrarius* (n = 1)	1 (1)	0	1	22 (2)	2	4	0
*Apodemus flavicollis* (n = 3)	0	0	0	31 (5)	0	5	0
*Apodemus sylvaticus* (n = 1)	0	0	0	16 (2)	0	2	0
Total *Apodemus* (n = 5)	1 (1)	0	1	69 (9)	2	11	0
Total rodents (n = 35)	405 (49)	22	71	405 (54)	38	92	10

Juvenile ticks, larvae and nymphs, were first subjected to DNA extraction. In order to increase the efficiency of molecular research, larvae of certain tick species were processed in pools, comprising 2–10 larvae from one host. Nymphs were processed individually. Genomic DNA was extracted from ticks/pools using Mini AX Tissue Spin DNA extraction kit (A&A Biotechnology, Gdańsk, Poland) in accordance with the manufacturer's protocol.

For the detection of DNA of the *Rickettsia*, genus-specific primers CS409 (5′-CCTATGGCTATTATGCTTGC-3′) and Rp1258 (5′-ATTGCAAAAAGTACAGTGAACA-3′) were used for amplification of a 750 bp fragment of the citrate synthase gene (*gltA*) (Roux et al. 1997) in modified condition as described by Kowalec et al. (2019).

For molecular screening of spirochaetes (*B. burgdorferi* s.l.) genus-specific primers: 132f/905r and 220f/824r (Wodecka et al. 2009) were used to amplify the bacterial *flaB* gene fragments (774 and 605 bp), respectively (Wodecka et al. 2009), in nested-PCR protocol in modified reaction conditions (Kowalec et al. 2017).

Nested-PCR targeting 18S rDNA was performed to detect DNA of *Babesia* spp. In the first reaction with the outer primers CRYPTO R/ CRYPTO F (Bonnet et al. 2007) a fragment of ca. 1200 bp long was amplified. For the second reaction primers Bab GR2/ Bab GF2 were used to amplify a ca. 550 bp fragment. DNA of *Babesia canis* was used as positive control. For both nested PCR protocols (for *B. burgdorferi* s.l. and *Babesia* spp.) 1.0 μl of the first reaction product was used as the template DNA for the nested reactions.

All ticks tested in the present study had to be treated as partially engorged as they were collected from hosts. To compare occurrence of pathogen DNA in ticks and host samples, host samples (blood) were also tested for the presence of the pathogen DNA. DNeasy Blood & Tissue Kit (Qiagen, USA) was used for extraction of genomic DNA from rodent blood samples. Identical PCR protocols as described for tick samples were used for testing of rodent samples.

To confirm correct classification of voles to the genera *Microtus* (*M. arvalis*, *M. agrestis*) and *Alexandromys* (*A. oeconomus*), amplification with primers L14729 + H15906arvic (Lebedev et al. 2007) and sequencing of a 900 bp fragment of *cytB* gene were applied on selected host samples as described in Lissovsky et al. (2018).

Reactions were performed in 20 μl volume, including 1 × PCR Dream Taq Green Buffer (Thermo Fisher Scientific Baltics UAB, Vilnius, Lithuania), 1U Dream Taq polymerase, 2 mM dNTP, 1 μM of each primer and 2 μl of the extracted DNA sample. Negative controls were performed with 2 μl of sterile water in the absence of template DNA. PCR products were visualized on 1.5% agarose gel stained with Midori Green Stain (Nippon Genetics Europe, Düren, Germany).

Selected PCR products obtained from ticks and host blood were sequenced by a private company (Genomed, Warsaw, Poland). Sequence alignments and analyses were carried out using BLAST-NCBI and MEGA X software (Kumar et al. 2018). Phylogenetic analyses were performed using the maximum likelihood method of tree-construction. The evolutionary model was chosen in accordance to the data (following implemented model test in MEGA X) and bootstrapped over 1000 randomly generated sample trees. Identical sequences obtained in the study were pooled for analysis. In case of *Borrelia* spp., the evolutionary history was inferred by using the maximum likelihood method and Hasegawa-Kishino-Yano model (Hasegawa et al. 1985). For *Babesia* sp. analyses, the evolutionary history was inferred by using the maximum likelihood method and Kimura 2-parameter model (Kimura, 1980). For the *cytB* gene fragment from rodents, the evolutionary history was inferred by using the maximum likelihood method and Hasegawa-Kishino-Yano model. For each analysis, initial tree(s) for the heuristic search were obtained automatically by applying Neighbor-Join and BioNJ algorithms to a matrix of pairwise distances estimated using the Maximum Composite Likelihood (MCL) approach, and then selecting the topology with superior log likelihood value. Additionally, phylogenetic analysis was performed with the Neighbor Joining and Minimum Evolutionary methods, and tree topologies were compared for a robust phylogeny (supplementary files).

### Statistical analysis

For the analysis of prevalence (% PCR-positive samples), we applied maximum likelihood techniques based on log linear analysis of contingency tables in the IBM SPSS v.21 software package. Factors such as: tick species (3 levels: *H*. *concinna*, *I. ricinus*, *D. reticulatus*), host genus [2 levels: *Apodemus* combined (*A. agrarius, A. flavicollis, A. sylvaticus*), *Microtus* + *Alexandromys* combined (*A. oeconomus*, *M. agrestis*)], and—only for nymphs—co-infections (3 levels: 0, lack of pathogen; 1, one pathogen; 2, two or three pathogens detected in a tick) were used in models with the presence or absence of pathogen DNA considered as a binary factor (0, 1). For each level of analysis in turn, beginning with the most complex model, involving all possible main effects and interactions, those combinations that did not contribute significantly to explaining variation in the data were eliminated in a stepwise fashion beginning with the highest level interaction (backward selection procedure). A minimum sufficient model was then obtained, for which the likelihood ratio of χ^2^ was not significant, indicating that the model was sufficient in explaining the data (Behnke et al. 2001; Bajer et al. 2005).

### Ethical statement

All of the procedures conducted on rodents were approved by the First Warsaw Local Ethics Committee for Animal Experimentation in Poland (ethical license number: 706/2015).

## Results

In total, 71 *H. concinna* samples (22 nymphs and 49 pools of larvae), 92 *I. ricinus* samples (38 nymphs and 54 pools of larvae) and 10 nymphs of *D. reticulatus* were screened for the presence of pathogens. The structure of the samples from certain host species is shown in Table 1.

The overall detection (% positive) of specific pathogen DNA in all samples (larvae pools + nymphs) differed between the examined tick species. DNA of *Rickettsia* spp. was found in four *D. reticulatus* nymphs, in 31.5% of *I. ricinus* samples (72.4% of larvae pools, 27.6% of nymphs), and the lowest prevalence was found in *H. concinna* (2.9%; only in two larvae pools) (*Rickettsia* spp. presence/absence × tick species: χ^2^ = 23.26, d.f. = 2, *P* < 0.001).

DNA of *B. burgdorferi* s.l. was detected in 67.4% of *I. ricinus* samples (64.8% of larvae pools, 71.7% of nymphs), in almost 40% of *H. concinna* (51% of larvae pools, 13.6% of nymphs) and in one *D. reticulatus* nymph (*B. burgdorferi* s.l. presence/absence × tick species: χ^2^ = 33.17, d.f. = 2, *P* < 0.001).

DNA of *Babesia* spp. was identified in more than 40% of *I. ricinus* samples (57.4% of larvae pools, 18.4% of nymphs) and in 7% of *H. concinna* samples (6.1% of larvae pools, 9.1% of nymphs) (*Babesia* spp. presence/absence × tick species: χ^2^ = 33.10, d.f. = 2, *P* < 0.001).

### Influence of host species/genus on pathogen detection in feeding ticks

#### *Haemaphysalis* concinna

Although *H. concinna* ticks were collected both from voles (*Microtus* and *Alexandromys*) and mice (*Apodemus*), pathogen DNA was detected only in *H. concinna* obtained from voles. In ticks collected from *A. oeconomus*, DNA of *Rickettsia* spp., *B. burgdorferi* s.l. and *Babesia* spp. was detected (in 2.9, 38.2 and 7.4% of samples, respectively). *Borrelia burgdorferi*-positive samples were also found in a few ticks collected from *M. agrestis*.

#### *Ixodes* ricinus

A similar prevalence of *Rickettsia* spp. positive samples was found in ticks collected from *Apodemus* spp. and *Microtus* + *Alexandromys* (36.4 and 30.9%, respectively). DNA of *Rickettsia* was detected in 31.6% samples of ticks collected from *A. oeconomus* and from two species of mice: *A. agrarius* (25% positive) and *A. flavicollis* (60% positive) (percentages were not significantly different).

The percentage of *B. burgdorferi-*positive samples in *I. ricinus* ticks was high, regardless of the rodent species from which ticks were collected (69.1% in tick samples from *Microtus* + *Alexandromys*, 54.6% in tick samples from *Apodemus* spp.). The DNA of *B. burgdorferi* s.l. was detected in all samples of ticks collected from the striped field mouse, *A. agrarius*. One *B. burgdorferi*-positive sample was found in ticks collected from the field voles *M. agrestis*, and two from yellow necked mice, *A. flavicollis*.

DNA of *Babesia* spp. was found in ticks collected from both voles (genera *Microtus* + *Alexandromys*) and *Apodemus* spp.; however, only in 9.1% of *I. ricinus* collected from mice and in 45.7% of ticks collected from voles (percentages not significantly different). Similar high percentages of *Babesia-*positive samples were detected in ticks collected from *A. oeconomus* (45.6%), *M. agrestis* (one sample, 50%) and in one sample also from *A. flavicollis* (20%) (percentages not significantly different). DNA of *Babesia* spp. was not detected in ticks obtained from *A. agrarius* and *A. sylvaticus*.

Among 10 *D. reticulatus* nymphs collected from *A. oeconomus*, one was positive for *Rickettsia* spp. and four for *B. burgdorferi* s.l.

### Molecular detection of pathogen DNA in rodents

Host species had no significant effect on the prevalence of *Babesia* infection in rodents; however, 33.1% (nine individuals) of *A. oeconomus*, one *M. agrestis* and one *A. flavicollis* were *Babesia*-positive. *Apodemus agrarius* and *A. sylvaticus* tested negative for *Babesia* infection. *Alexandromys oeconomus* was the only rodent species in which DNA of *B. burgdorferi* s.l. was detected in blood (in 17.9%, five individuals). DNA of *Rickettsia* was not detected in rodent blood samples.

### Detection of pathogen DNA in ticks from infected and non-infected rodents

Host infection status had an effect on detection of *B. burdorferi* s.l. in ticks depending on tick stadium (*B. burgdorferi* s.l*.* tick infection × host infection × tick stadium: χ^2^ = 8.45, d.f. = 1, P = 0.01). DNA of *B. burgdorferi* s.l was detected in 34.1% of nymphs and 65.9% of larvae pools collected from *Borrelia*-positive rodents, in comparison with 47.6% of nymphs and 52.4% of larvae collected from *Borrelia*-negative rodents.

Most larvae pools obtained from *Babesia*-infected rodents were positive (79.1%) compared with 53.1% of positive larvae pools collected from uninfected hosts (percentages not significantly different). However, DNA of *B. microti* was detected in 46.9% of nymphs collected from *B. microti*-negative rodents and in 20.9% of nymphs collected from *B. microti*-positive rodents.

### Co-infections in nymphs

Altogether, DNA of one pathogen species was detected in 22.7, 60.5 and 50% of *H. concinna, I. ricinus* and *D. reticulatus* nymphs, respectively. Co-infections of two and three pathogens were found in 23.7% of *I. ricinus* nymphs. No co-infections were detected in *H. concinna* nor in *D. reticulatus* nymphs (tick species × co-infections: χ^2^ = 29.25, d.f. = 1, *P* < 0.001).

### Molecular identification of pathogen species/genotypes

**Fig. 1:**
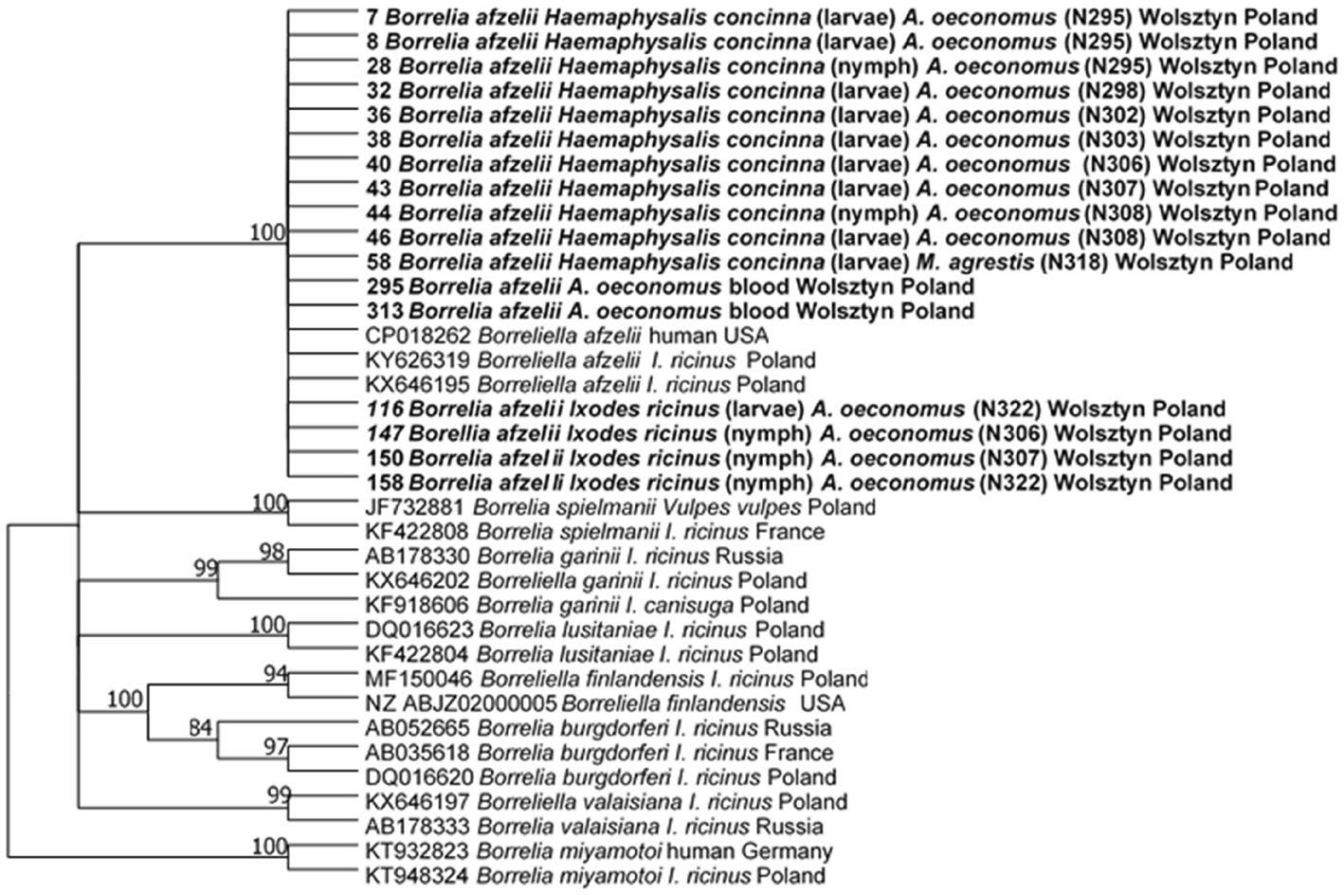
Molecular phylogenetic analysis of *flaB* of *Borelia burgdorferi* s.l. (605 bp), showing the tree with the highest log likelihood (− 1677.91). The percentage of trees in which the associated taxa clustered together is shown next to the branches. This analysis involved 36 nucleotide sequences. All positions containing gaps and missing data were eliminated (complete deletion option). In total, there were 529 positions in the final dataset

Twenty-two *Borrelia*-positive PCR products were sequenced: 11 from *H. concinna* (from two nymphs and nine larvae pools; 39.4% of 28 positive samples), nine from *I. ricinus* (one larvae pool and eight nymphs; 14.5% of 62 positive samples) and two from *A. oeconomus* voles. All sequences displayed the highest identity (99–100%) to *Borrelia afzelii* (GenBank accession number KX646195). The phylogenetic tree, incorporating 17 sequences obtained in this study and 19 reference sequences from GenBank, is presented in Fig. 1. The tree topology showed that sequences obtained from two tick species and voles clustered on one separate branch, as expected from BLAST analysis, constituting the *B. afzelii* clade. Trees constructed using Minimum Evolutionary and Neighbor Joining models showed similar topology to the maximum likelihood analysis (Supplementary file 1).

Nine *Babesia* spp. PCR products from *I. ricinus* larvae pools (23.7% of 28 positive samples) and four *Babesia* spp. PCR products from *H. concinna* (one nymph and three larvae pools; 80% of five samples) were successfully sequenced. Eleven obtained sequences (all from *I. ricinus* and two from *H. concinna*) were essentially identical (identity above 99.9%) to the sequence of *B. microti* Jena genotype (EF413181). However, two *Babesia* sequences obtained from *H. concinna* displayed the highest similarity (97.4 and 100%, respectively) to undescribed *Babesia* species from *H. concinna*, Russia (KJ486560) and only 93.7% identity with *B. microti* (KC821597).

**Fig. 2:**
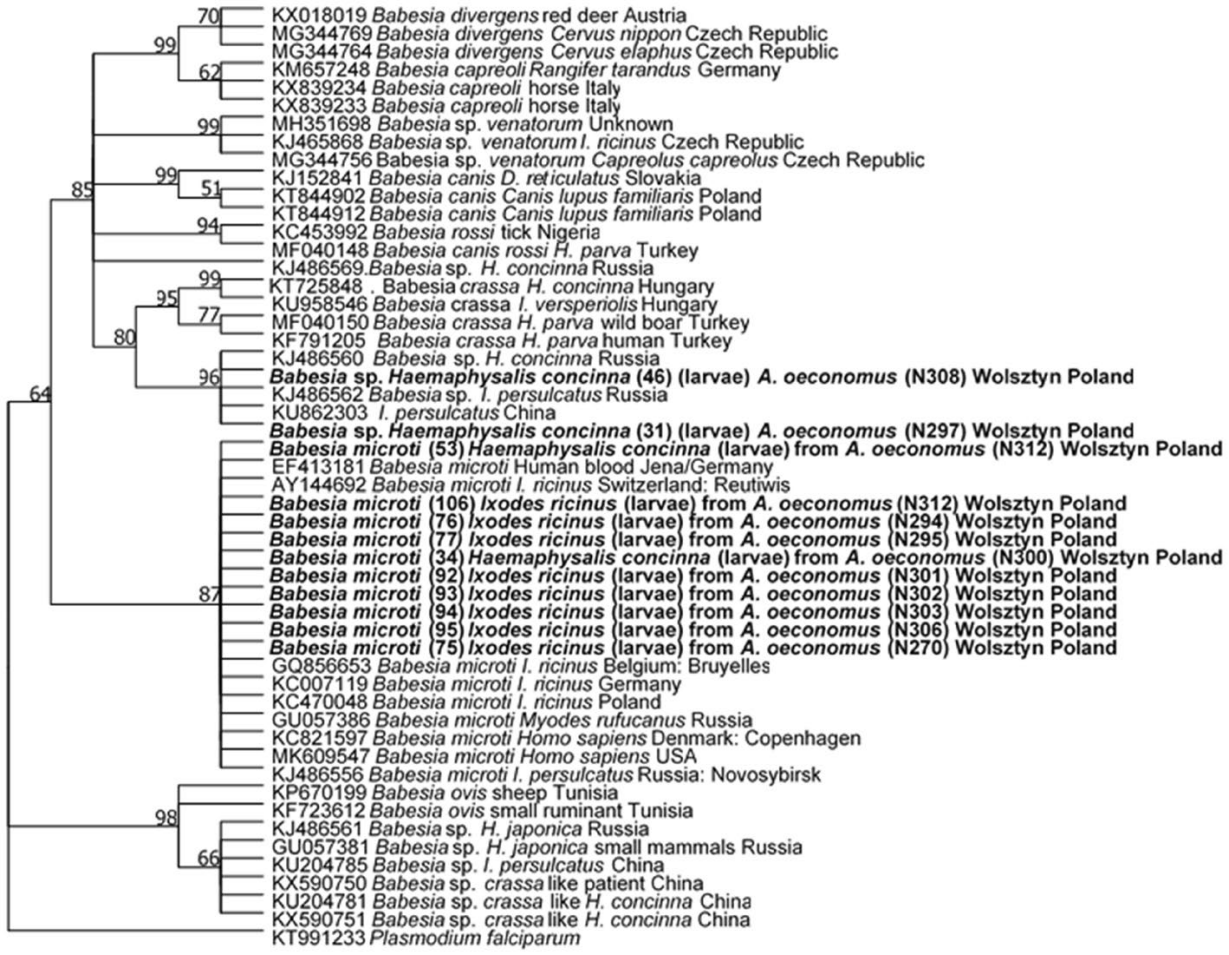
Molecular phylogenetic analysis of 18S rDNA of *Babesia* spp. (550 bp), showing the tree with the highest log likelihood (− 2819.70). The percentage of trees in which the associated taxa clustered together is shown next to the branches. A discrete Gamma distribution was used to model evolutionary rate differences among sites [five categories (+ *G*, parameter = 2.1193)]. This analysis involved 52 nucleotide sequences. In total, there were 462 positions in the final dataset

A representative tree for 18S rDNA sequences, obtained using the maximum likelihood method and a Kimura 2-parameter model is presented in Fig. 2. Our *B. microti* sequences from *I. ricinus* and *H. concinna* grouped together with numerous *B. microti* zoonotic isolates from ticks, rodents and humans (Fig. 2). Two unidentified *Babesia* sequences from *H. concinna* grouped separately with a few undescribed *Babesia* sequences from *I. persulcatus* and *H. concinna* from Russia (Rar et al. 2014) and China (Fig. 2). Interestingly, this group of sequences was the most similar (sister group) to ovine piroplasm *B. crassa* (95.7% similarity) and to *B. crassa*-like group pathogenic for humans (Jia et al. 2018). Trees constructed using Minimum Evolutionary and Neighbor Joining models showed similar topology to the maximum likelihood analysis (Supplementary file 2).

For *Rickettsia* spp. only three sequences of the 750-bp fragment of the citrate synthase gene (*gltA*) were obtained. Two *Rickettsia* sequences from *I. ricinus* (one larvae pool and one nymph) displayed the highest identity (99.6 and 97.5%, respectively) with *R. helvetica* from *I. ricinus* from Serbia (MH618386). A sequence from a *D. reticulatus* nymph was identified as *R. raoulti* (99.4% similarity to sequences MN388798 (from *Carios vespertilionis*, China), MT178334 (from *Dermacentor nuttalli*, China) and KU310589 (from *D. reticulatus*, Russia). Sequencing of *Rickettsia* PCR products from *H. concinna* was unsuccessful.

### Molecular identification of rodent species

**Fig. 3:**
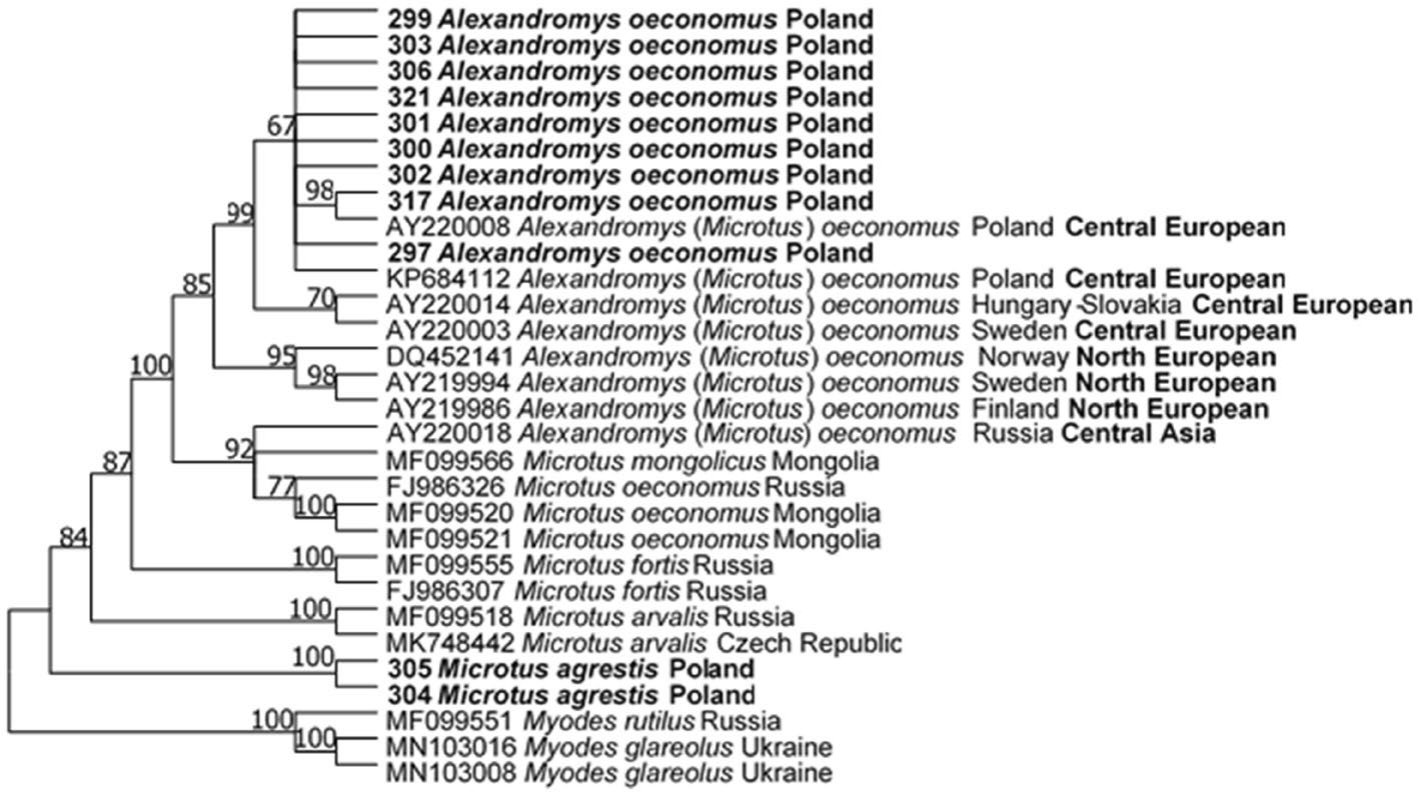
Molecular phylogenetic analysis of *cytB* gene (900 bp), showing the tree with the highest log likelihood (− 3109.99). The percentage of trees in which the associated taxa clustered together is shown next to the branches. This analysis involved 30 nucleotide sequences. In total, there were 871 positions in the final dataset

Eleven of 16 positive PCR products (900 bp *cytB* fragment) were sequenced for differentiation of *Microtus* and *Alexandromys* voles. Nine sequences from *A. oeconomus* displayed the highest identity (98.2–99.7%) with sequence AY220010 representing *M. oeconomus* (*A. oeconomus*) from Poland and clustered with a Central European *A. oeconomus* allopatric phylogroup (Brunhoff et al. 2003; Galbreath and Cook, 2004; Iwasa et al. 2009) (Fig. 3). Two sequences were the most similar (99.0–99.9%) to *M. agrestis* from Poland (KF218938). Trees constructed using Minimum Evolutionary and Neighbor Joining models showed similar topology to the maximum likelihood analysis (Supplementary file 3).

## Discussion

Present study revealed that DNA of three tick-borne pathogens—*Babesia*, *B. burgdorferi* s.l. and *Rickettsia* spp.—can be detected in high percentage of juvenile ticks feeding on rodents, thus confirming the pivotal role of rodents as the source of blood meal and/or infection for three species of ticks. Present study provided also the first results on the detection of the pathogen DNA in juvenile *H. concinna* ticks from the area of Poland. Additionally, *A. oeconomus* seems to be the host of the highest significance, due to high prevalence of *Babesia* and *Borrelia* either in ticks collected from this host species or in hosts themselves. One of the most important findings is the detection of new (undescribed) *Babesia* species in *H. concinna,* similar to *Babesia* found in the Far East (Russia and China) and Slovakia related to *B. crassa*, probably representing species of piroplasm specific for *H. concinna*.

In the present study, substantial numbers of juvenile *I. ricinus* (about 440) and *H. concinna* (427) ticks were tested and DNA of all three tick-borne pathogens was identified both in larvae pools and in nymphs of both species. Among 10 *D. reticulatus*, only five were positive (one for *B. afzelii*, four for *Rickettsia* [2 × *R. raoulti*]).

Among juvenile *H. concinna* and *I. ricinus* feeding on rodents, the most common pathogen was *B. burgdorferi* s.l. (mostly *B. afzelii*) found in almost 40% of *H. concinna* and 67% of *I. ricinus*. Interestingly, DNA of *B. burgdorferi* s.l*.* was found not only in ticks collected form positive rodents but also in ticks collected from negative hosts, and from hosts representing different genera (*Apodemus*, *Alexandromys* and *Microtus*). However, we tested rodent blood samples for *Borrelia* presence, and occurrence of *B. burgdorferi* in blood can be short in comparison to its persistence in other host tissues (Liang et al. 2020)—some positive rodents could have tested false negative based on blood samples. Taking into account the high percentage of *B. burgdorferi*-positive ticks, and especially high prevalence in *I. ricinus* nymphs and *H. concinna* larvae, we may conclude that this is the most common tick-transmitted pathogen circulating between ticks and rodents, and possessing the highest public health threat. Thus, this is the first study to demonstrate that also *H. concinna* ticks feeding on infected rodents may play a role as vectors of *B. burgdorferi* s.l., particularly for *B. afzelii* (all sequenced PCR products were *B. afzelii*). There is limited data on spirochaetes detection in *H. concinna*. DNA of *B. afzelii* was detected in *H. concinna* nymphs in Hungary (Rigo et al. 2011). Two *Borrelia* species were detected in adult ticks: *B. garinii* in un-fed, host-seeking *H. concinna* from southeastern China (Chu et al. 2008) and *B. miyamotoi* in one from 36 examined *H. concinna* in northeastern China (Jiang et al. 2018). From Far East Russia, only 14 of 481 examined *H. concinna* ticks were *B. burgdorferi* s.l.-positive (Pukhovskaya et al. 2019). However, in many other studies *H. concinna* ticks collected from vegetation or rodents (Central Europe and China) tested negative for *Borrelia* infections (Kahl et al. 1992; Blaschitz et al. 2008; Wang et al. 2019; Heglasová et al. 2020).

Our result (67.4% of positive samples of *B. burgdorferi* s.l. in *I. ricinus* instars) was definitely higher than prevalence of *Borrelia* spp. in *I. ricinus* questing ticks examined in other studies in Poland. Prevalence of *B. burgdorferi* s.l. in questing adult *I. ricinus* was about 12% in urban parks in Warsaw, as well as in natural forests near Białowieża (Kowalec et al. 2017). In northeastern Poland, prevalence of *B. burgdorferi* s.l. was about 13% in questing *I. ricinus* nymphs (Kubiak et al. 2019) and almost 45% in pooled unfed nymphs in Lower Silesia, western Poland (Kiewra et al. 2014).

The second most abundant pathogen was *Rickettsia*—about 28% of juvenile *H. concinna* and 31% of juvenile *I. ricinus* ticks tested positive (and four out of 10 *D. reticulatus*, 40%). A similar and high percentage of detection of *Rickettsia* spp. DNA in ticks regardless of the tick species and stadium, or host species from which ticks were collected, is in agreement with the endosymbiont status of these bacteria in ticks (circulating in tick populations, independent on external sources of infection; Parola et al. 2013). Genotyping revealed the presence of typical tick-specific species: *R. raoultii* in *D. reticulatus* and *R. helvetica* in *I. ricinus* (Chmielewski et al. 2009; Stańczak et al. 2016; Kowalec et al. 2019); unfortunately, species of *Rickettsia* from *H. concinna* could not be determined (unsuccessful sequencing). No *Rickettsia* DNA was found in rodents. There are not enough data on vertebrates as a reservoir of these bacteria (Tomassone et al. 2018) and the role of rodents as a source of these pathogens needs more attention.

Although *Babesia* infection was quite common among three host species (17–40% in *A. flavicollis*, *A. eoconomus* and *M. agrestis*), detection of this parasite’s DNA differed profoundly between tick species: it was not detected in the 10 *D. reticulatus*, but was detected in about 7% of *H. concinna* and in 41% (18% nymphs and 57% larvae pools) of *I. ricinus*. Apparently, detection of *B. microti* in ticks, tick hosts and rodents, was highly associated: up to 80% of *I. ricinus* larvae pools originated from infected rodents (*A. oeconomus*, *A. flavicollis* and *M. agrestis*) tested positive. However, we obtained reversed relationship with the percentage of *Babesia*-infected *I. ricinus* nymphs: twice higher in nymphs collected from negative rodents than from positive ones. Nevertheless, *B. microti* is abundant among rodents, especially voles (Karbowiak et al. 2005; Tołkacz et al. 2017; Dwużnik et al. 2019b) and surely voles are the main source of infections for juvenile ticks feeding on them. The presence of DNA of a zoonotic strain of *B. microti* in *H. concinna* is a novel finding (Zhou et al. 2014); however, the vector competence of *H. concinna* for this *Babesia* species must be verified in experimental transmission studies (Gray et al. 2019) before a final conclusion can be drawn.

The new *Babesia* species seems to be quite specific for *H. concinna* because this genotype was found in this tick species both in Central Europe—in Poland (present study), Slovakia (Hamšíková et al. 2016), Hungary (Flaisz et al. 2017)—and in the Far East in Russia (Rar et al. 2014), although it was also found in *I. persulcatus* in China (Rar et al. 2014). Interestingly, in China *B. crassa*-like, which was found in *H. concinna* ticks, was also pathogenic for humans (Jia et al. 2018). High similarity of *Babesia* sp. found in the current study to the abovementioned *B. crassa*-like from humans and *H. concinna* in China (phylogenetic trees) brings attention to pathogenic potential of this *Babesia* species—a possible new health threat in Central Europe.

Rodent species trapped in our study have a wide geographical range and occur commonly in Eurasia, including the western region of Poland. They are abundant in various habitats (natural, urban, semi-urban; *Apodemus* spp.), in fallow land, wetland, river basin (*Alexandromys*, *Microtus*) (Lubicz-Niezawitowski 1933; Paziewska et al. 2010; Gortat et al. 2014; Dwuznik et al. 2017; Welc-Faleciak et al. 2008). After molecular and phylogenetic analyses, we can confirm the new taxonomic status of the root vole *A. oeconomus*, previously a subgenus of *Microtus*, now elevated to full generic rank (Lissovsky and Obolenskaya 2011; Lissovsky et al. 2018; Zorenko and Atanasov 2018). Phylogenetic analysis confirmed not only the division between *Microtus* and *Alexandromys*, but also separated the allopatric phylogroups among *A. oeconomus* species (Brunhoff et al. 2003; Galbreath and Cook, 2004; Iwasa et al. 2009).

*Alexandromys oeconomus* is apparently a very important host for all three tick species and *B. afzelii* and *B. microti* pathogens. To our best knowledge, this is a first report of juvenile *H. concinna* feeding on *A. oeconomus*. Juvenile *H. concinna* were previously collected from medium size mammals, like roe deer, goats and sheep (Hornok et al. 2016), birds (Flaisz et al. 2017), European ground squirrel, *Spermophilus citellus* (Radulović et al. 2017) and even lizards (Avila and Morando 2003). However, data on rodents as the host species for *H. concinna* are scarce (Dwużnik et al. 2019a; Heglasová et al. 2020). *Haemaphysalis concinna* is still considered a rare tick species, occurring in isolated limited locations, so any new data on hosts for juvenile stages uncover new knowledge on their host reservoir and the possibility of pathogens circulation.

## Conclusions

Our study showed differences in prevalence of various pathogens between three tick species. For the first time in Poland we detected a new species of *Babesia* sp. specific to *H. concinna.* Full identification of the new, unnamed *Babesia* species (host reservoir, pathogenicity, microbiological assessment, geographical range, etc.) needs further investigation. Also, we highlighted that morphological features may not be sufficient for correct identification of rodents from *Alexandromys* and *Microtus* genera.

## Supplementary Information

Below is the link to the electronic supplementary material.

Supplementary file1 (DOCX 70 kb)

Supplementary file2 (DOCX 69 kb)

Supplementary file3 (DOCX 80 kb)

Supplementary file4 (DOCX 81 kb)

Supplementary file5 (DOCX 58 kb)

Supplementary file6 (DOCX 58 kb)

## Data Availability

All data generated or analysed during this study are included in this published article.
