# Supplementary material for: Pathogens detected in the tick Haemaphysalis concinna in Western Poland: known and unknown threats
Source: Exp Appl Acarol. 2021 Aug 11;84(4):769–83. doi: 10.1007/s10493-021-00647-x (PMC8367898; doi:10.1007/s10493-021-00647-x)
Supplement: Supplementary file 1 — Supplementary file1 (DOCX 70 kb) [file 10493_2021_647_MOESM1_ESM.docx]

**7 *Borrelia afzelii Haemaphysalis concinna* (larvae) *A. oeconomus* Wolsztyn Poland**

**8 *Borrelia afzelii Haemaphysalis concinna* (larvae) *A. oeconomus* Wolsztyn Poland**

**28 *Borrelia afzelii Haemaphysalis concinna* (nymph) *A. oeconomus* Wolsztyn Poland**

**32 *Borrelia afzelii Haemaphysalis concinna* (larvae) *A. oeconomus* Wolsztyn Poland**

**36 *Borrelia afzelii Haemaphysalis concinna* (larvae) *A. oeconomus* Wolsztyn Poland**

**38 *Borrelia afzelii Haemaphysalis concinna* (larvae) *A. oeconomus* Wolsztyn Poland**

**40 *Borrelia afzelii Haemaphysalis concinna* (larvae) *A. oeconomus* Wolsztyn Poland**

**43 *Borrelia afzelii Haemaphysalis concinna* (larvae) *A. oeconomus* Wolsztyn Poland**

**44 *Borrelia afzelii Haemaphysalis concinna* (nymph) *A. oeconomus* Wolsztyn Poland**

**46 *Borrelia afzelii Haemaphysalis concinna* (larvae) *A. oeconomus* Wolsztyn Poland**

**58 *Borrelia afzelii Haemaphysalis concinna* (larvae) *M. agrestis* Wolsztyn Poland**

**295 *Borrelia afzelii A. oeconomus* (N295) blood Wolsztyn Poland**

**313 *Borrelia afzelii A. oeconomus* (N313) blood Wolsztyn Poland**

CP018262 Borreliella afzelii human USA

KY626319 *Borreliella afzelii* *I. ricinus* Poland

KX646195 *Borreliella afzelii I. ricinus* Poland

**116 *Borrelia afzelii Ixodes ricinus* (larvae) *A,oeconomus* (N322) Wolsztyn Poland**

**147 *Borellia afzelii Ixodes ricinus* (nymph) *A. oeconomus* (N306) Wolsztyn Poland**

**150 *Borrelia afzelii Ixodes ricinus* (nymph) *A. oeconomus* (N307) Wolsztyn Poland**

**158 *Borrelia afzelii Ixodes ricinus* (nymph) *A. oeconomus* (N322) Wolsztyn Poland**

JF732881 *Borrelia spielmanii Vulpes vulpes* Poland

KF422808 *Borrelia spielmanii I. ricinus* France

AB178330 *Borrelia garinii I. ricinus* Russia

KX646202 *Borreliella garinii I. ricinus* Poland

KF918606 *Borrelia garinii I. canisuga* Poland

DQ016623 *Borrelia lusitaniae I. ricinus* Poland

KF422804 *Borrelia lusitaniae I. ricinus* Poland

MF150046 *Borreliella finlandensis I. ricinus* Poland

NZ ABJZ02000005 *Borreliella finlandensis* USA

AB052665 *Borrelia burgdorferi I. ricinus* Russia

AB035618 *Borrelia burgdorferi I. ricinus* France

DQ016620 *Borrelia burgdorferi I. ricinus* Poland

KX646197 *Borreliella valaisiana I. ricinus* Poland

AB178333 *Borrelia valaisiana I. ricinus* Russia

KT932823 *Borrelia miyamotoi* human Germany

KT948324 *Borrelia miyamotoi I. ricinus* Poland

100

98

95

100

98

94

100

100

100

100

52

55

100

57

65

66

67

71

73

75

75

79

86

86

88

94

100

**Supplementary file 1a** Molecular phylogenetic analysis of *flaB* of *Borelia burgdorferi* s.l. (605 bp)

The evolutionary history was inferred using the **Minimum Evolution method** (Rzhetsy and Nei,1992). The optimal tree is shown. The percentage of replicate trees in which te associated taxa clustered together in the bootstrap test (1000 replicates) are shown next to the branches (Felsenstein, 1985). The evolutionary distances were computed using the Maximum Composite Likelihood method (Tamura et al. 2004) and are in the units of the number of base substitutions per site. The ME tree was searched using the Close-Neighbor-Interchange (CNI) algorithm (Nei and Kumar, 2004) at a search level of 1. The Neighbor-joining algorithm (Saitou and Nei, 1987) was used to generate the initial tree. This analysis involved 36 nucleotide sequences. All ambiguous positions were removed for each sequence pair (pairwise deletion option). There were a total of 541 positions in the final dataset. Evolutionary analyses were conducted in MEGA X (Kumar et al. 2018).
