# Supplementary material for: Pathogens detected in the tick Haemaphysalis concinna in Western Poland: known and unknown threats
Source: Exp Appl Acarol. 2021 Aug 11;84(4):769–83. doi: 10.1007/s10493-021-00647-x (PMC8367898; doi:10.1007/s10493-021-00647-x)
Supplement: Supplementary file 3 — Supplementary file3 (DOCX 80 kb) [file 10493_2021_647_MOESM3_ESM.docx]

KU862303 *I. persulcatus* China

***Babesia* sp. *Haemaphysalis concinna* (31) (larvae) *A. oeconomus* (N297) Wolsztyn Poland**

KJ486560 *Babesia* sp. *H. concinna* Russia

KJ486562 *Babesia* sp. *I. persulcatus* Russia

***Babesia* sp. *Haemaphysalis concinna* (46) (larvae) *A. oeconomus* (N308) Wolsztyn Poland**

KJ486569 *Babesia* sp. *H. concinna* Russia

KT725848 *Babesia* *crassa* *H. concinna* Hungary

*KU958546 Babesia crassa I. versperiolis* Hungary

MF040150 *Babesia crassa* *H. parva* wild boar Turkey

KF791205 *Babesia crassa* *H. parva* human Turkey

KT844902 *Babesia canis Canis lupus familiaris* Poland

KJ152841 *Babesia canis D. reticulatus* Slovakia

KT844912 *Babesia canis Canis lupus familiaris* Poland

KC453992 *Babesia rossi* tick Nigeria

MF040148 *Babesia canis rossi Haemaphysalis parva* Turkey

KJ465868 *Babesia* sp. *venatorum I. ricinus* Czech Republic

MH351698 *Babesia* sp*. venatorum* Unknown

MG344756 *Babesia* sp. *venatorum Capreolus capreolus* Czech Republic

KX018019 *Babesia divergens* red deer Austria

MG344769 *Babesia divergens* *Cervus nippon* Czech Republic

MG344764 *Babesia divergens* *Cervus elaphus* Czech Republic

KM657248 *Babesia capreoli* *Babesia capreoli* *Rangifer tarandus* Germany

KX839234 *Babesia capreoli* horse Italy

KX839233 *Babesia capreoli* horse Italy

***Babesia microti* (53) *Haemaphysalis concinna* (larvae) from *A. oeconomus* (N312)Wolsztyn Poland**

KC007119 *Babesia microti I. ricinus* Germany

KC470048 *Babesia microti I. ricinus* Poland

***Babesia microti* (75) *Ixodes ricinus* (larvae) from *A. oeconomus* (N270) Wolsztyn Poland**

***Babesia microti* (95) *Ixodes ricinus* (larvae) from *A. oeconomus* (N306) Wolsztyn Poland**

GQ856653 *Babesia microti I. ricinus* Belgium: Bruyelles

***Babesia microti* (92) *Ixodes ricinus* (larvae) from *A. oeconomus* (N301) Wolsztyn Poland**

***Babesia microti* (93) *Ixodes ricinus* (larvae) from *A. oeconomus* (N302) Wolsztyn Poland**

***Babesia microti* (94) *Ixodes ricinus* (larvae) from *A. oeconomus* (N303) Wolsztyn Poland**

***Babesia microti* (34) *Haemaphysalis concinna* (larvae) from *A. oeconomus* (N300) Wolsztyn Poland**

***Babesia microti* (77) *Ixodes ricinus* (larvae) from *A. oeconomus* (N295) Wolsztyn Poland**

***Babesia microti* (76) *Ixodes ricinus* (larvae) from A. oeconomus (N294) Wolsztyn Poland**

***Babesia microti* (106) *Ixodes ricinus* (larvae) from *A. oeconomus* (N312) Wolsztyn Poland**

AY144692 *Babesia microti I. ricinus* Switzerland: Reutiwis

EF413181 *Babesia microti* Human blood Jena/Germany

GU057386 *Babesia microti Myodes rufucanus* Russia

KC821597 *Babesia microti Homo sapiens* Denmark: Copenhagen

MK609547 *Babesia microti Homo sapiens* USA

KJ486556 *Babesia microti* *I. persulcatus* Russia: Novosybirsk

GU057381 *Babesia* sp*. H. japonica* small mammals Russia

KU204781 *Babesia* sp. *crassa* like *H. concinna* China

KU204785 *Babesia* sp. *I. persulcatus* China

KX590750 *Babesia* sp. *crassa* like patient China

KF723612 *Babesia ovis* small ruminant Tunis *Babesia crassa* like *I. persulcatus* China

KP670199 *Babesia ovis* sheep Tunisia

KJ486561 Babesia sp. *H. japonica* Russia

KX590751 *Babesia* sp*. crassa* like *H. concinna* China

KT991233 *Plasmodium falciparum*

65

100

77

51

94

82

50

50

79

94

92

93

58

85

78

76

86

63

90

**Supplementary file 2a** Molecular phylogenetic analysis of 18S rDNA of *Babesia* spp. (550bp)

The evolutionary history was inferred using the **Minimum Evolution method** (Rzhetsky and Nei, 1992). The optimal tree is shown. The percentage of replicate trees in which the associated taxa clustered together in the bootstrap test (1000 replicates) are shown next to the branches (Felsenstein, 1985). The evolutionary distances were computed using the Maximum Composite Likelihood method (Tamura et al. 2004) and are in the units of the number of base substitutions per site. The ME tree was searched using the Close-Neighbor-Interchange (CNI) algorithm (Nei and Kumar, 2000) at a search level of 1. The Neighbor-joining algorithm (Saitou, 1987) was used to generate the initial tree. This analysis involved 52 nucleotide sequences. All ambiguous positions were removed for each sequence pair (pairwise deletion option). There were a total of 462 positions in the final dataset. Evolutionary analyses were conducted in MEGA X (Kumar et al. 2018).
