# Supplementary material for: Pathogens detected in the tick Haemaphysalis concinna in Western Poland: known and unknown threats
Source: Exp Appl Acarol. 2021 Aug 11;84(4):769–83. doi: 10.1007/s10493-021-00647-x (PMC8367898; doi:10.1007/s10493-021-00647-x)
Supplement: Supplementary file 4 — Supplementary file4 (DOCX 81 kb) [file 10493_2021_647_MOESM4_ESM.docx]

MG344769 *Babesia divergens* *Cervus nippon* Czech Republic

MG344764 *Babesia divergens* *Cervus elaphus* Czech Republic

KX018019 *Babesia divergens* red deer Austria

KX839234 *Babesia capreoli* horse Italy

KM657248 *Babesia capreoli* *Rangifer tarandus* Germany

KX839233 *Babesia capreoli* horse Italy

KJ465868 Babesia sp. venatorum strain IR146/Czech 18S ribosomal RNA gene partial sequence

MH351698 *Babesia* sp*. venatorum* Unknown

MG344756 *Babesia* sp. *venatorum Capreolus capreolus* Czech Republic

KT844902 *Babesia canis Canis lupus familiaris* Poland

KJ152841 *Babesia canis D. reticulatus* Slovakia

KT844912 *Babesia canis Canis lupus familiaris* Poland

KJ486569 *Babesia* sp. *H. concinna* Russia

KT725848 Babesia *crassa* *H. concinna* Hungary

KU958546 *Babesia crassa* *I. versperiolis* Hungary

MF040150 *Babesia crassa* *H. parva* wild boar Turkey

KF791205 *Babesia crassa* *H. parva* human Turkey

***Babesia* sp. *Haemaphysalis concinna* (46) (larvae) *A. oeconomus* (N308) Wolsztyn Poland**

KJ486562 *Babesia* sp. *I. persulcatus* Russia

KJ486560 *Babesia* sp. *H. concinna* Russia

KU862303 *I. persulcatus* China

***Babesia* sp. *Haemaphysalis concinna* (31) (larvae) *A. oeconomus* (N297) Wolsztyn Poland**

MK609547 *Babesia microti* *Homo sapiens* USA

KJ486556 *Babesia microti I. persulcatus* Russia: Novosybirsk

KC821597 *Babesia microti Homo sapiens* Denmark: Copenhagen

GU057386 *Babesia microti Myodes rufucanus* Russia

***Babesia microti* (53) *Haemaphysalis concinna* (larvae) from *A. oeconomus* (N312) Wolsztyn Poland**

EF413181 *Babesia microti* Human blood Jena/Germany

AY144692 *Babesia microti I. ricinus* Switzerland: Reutiwis

***Babesia microti* (106) *Ixodes ricinus* (larvae) from *A. oeconomus* (N312) Wolsztyn Poland**

***Babesia microti* (76) *Ixodes ricinus* (larvae) from *A. oeconomus* (N294) Wolsztyn Poland**

**Babesia microti (77) Ixodes ricinus (larvae)from A. oeconomus (N295) Wolsztyn Poland**

***Babesia microti* (34) *Haemaphysalis concinna* (larvae) from *A. oeconomus* (N300) Wolsztyn Poland**

***Babesia microti* (92) *Ixodes ricinus* (larvae) from *A. oeconomus* (N301) Wolsztyn Poland**

***Babesia microti* (93) *Ixodes ricinus* (larvae)from *A. oeconomus* (N302) Wolsztyn Poland**

***Babesia microti* (94) *Ixodes ricinus* (larvae) from *A. oeconomus* (N303) Wolsztyn Poland**

***Babesia microti* (95) *Ixodes ricinus* (larvae) from *A. oeconomus* (N306) Wolsztyn Poland**

***Babesia microti* (75) *Ixodes ricinus* (larvae) from *A. oeconomus* (N270) Wolsztyn Poland**

GQ856653 *Babesia microti I. ricinus* Belgium: Bruyelles

KC007119 *Babesia microti I. ricinus* Germany

KC470048 *Babesia microti I. ricinus* Poland

MF040148 *Babesia canis rossi H. parva* Turkey

KC453992 *Babesia rossi* tick Nigeria

GU057381 *Babesia* sp*. H. japonica* small mammals Russia

KU204781 *Babesia* sp. *crassa* like *H. concinna* China

KU204785 *Babesia* sp. *I. persulcatus* China

KX590750 *Babesia crassa* like *I. persulcatus* China

KF723612 *Babesia ovis* small ruminant Tunisia

KP670199 *Babesia ovis* sheep Tunisia

KJ486561 Babesia sp. *H. japonica* Russia

KX590751 *Babesia sp. crassa* like *H. concinna* China

KT991233 *Plasmodium falciparum*

67

100

92

98

98

96

53

53

85

97

98

96

99

99

96

96

82

82

94

77

51

81

81

56

87

**Supplementary file 2b** Molecular phylogenetic analysis of 18S rDNA of *Babesia* spp. (550bp)

The evolutionary history was inferred using the **Neighbor-Joining method** (Saitou, 1987). The optimal tree is shown. The percentage of replicate trees in which the associated taxa clustered together in the bootstrap test (1000 replicates) are shown next to the branches (Felsenstein, 1985). The evolutionary distances were computed using the Maximum Composite Likelihood method (Tamura et al. 2004) and are in the units of the number of base substitutions per site. This analysis involved 52 nucleotide sequences. All ambiguous positions were removed for each sequence pair (pairwise deletion option). There were a total of 462 positions in the final dataset. Evolutionary analyses were conducted in MEGA X (Kumar et al. 2018).
