# Supplementary material for: Pathogens detected in the tick Haemaphysalis concinna in Western Poland: known and unknown threats
Source: Exp Appl Acarol. 2021 Aug 11;84(4):769–83. doi: 10.1007/s10493-021-00647-x (PMC8367898; doi:10.1007/s10493-021-00647-x)
Supplement: Supplementary file 5 — Supplementary file5 (DOCX 58 kb) [file 10493_2021_647_MOESM5_ESM.docx]

**317 *Alexandromys oeconomus* Poland**

AY220008 *Alexandromys* (*Microtus*) *oeconomus* Poland **Central European**

**306 *Alexandromys oeconomus* Poland**

**321** ***Alexandromys oeconomus* Poland**

**300** ***Alexandromys oeconomus* Poland**

**301** ***Alexandromys oeconomus* Poland**

**302** ***Alexandromys oeconomus* Poland**

**299** ***Alexandromys oeconomus* Poland**

**303** ***Alexandromys oeconomus* Poland**

**297** ***Alexandromys oeconomus* Poland**

KP684112 *Alexandromys* (*Microtus*) *oeconomus* Poland **Central European**

AY220014 *Alexandromys* (*Microtus*) *oeconomus* Hungary-Slovakia **Central European**

AY220003 *Alexandromys* (*Microtus*) *oeconomus* Sweden **Central European**

DQ452141 *Alexandromys* (*Microtus*) *oeconomus* Norway **North European**

AY219994 *Alexandromys* (*Microtus*) *oeconomus* Sweden **North European**

AY219986 *Alexandromys* (*Microtus*) *oeconomus* Finland **North European**

MF099566 *Microtus mongolicus* Mongolia

AY220018 *Microtus mogolicus* Russia

FJ986326 *Alexandromys* (*Microtus*) *oeconomus* Russia

MF099520 *Alexandromys* (*Microtus*) *oeconomus* Mongolia

MF099521 *Alexandromys* (*Microtus*) *oeconomus* Mongolia

MF099555 *Microtus fortis* Russia

FJ986307 *Microtus fortis* Russia

**305 *Microtus agrestis* Poland**

**304** ***Microtus agrestis* Poland**

MF099518 *Microtus arvalis* Russia

MK748442 *Microtus arvalis* Czech Republic

MF099551 *Myodes rutilus* Russia

MN103016 *Myodes glareolus* Ukraine

MN103008 *Myodes glareolus* Ukraine

100

100

100

100

100

53

86

100

70

99

51

97

99

100

90

99

51

99

66

62

**Supplementary file 3a** Molecular phylogenetic analysis of *cytB* gene (900bp)

The evolutionary history was inferred using the **Minimum Evolution method** (Rzhetsky and Nei, 1992). The optimal tree is shown. The percentage of replicate trees in which the associated taxa clustered together in the bootstrap test (1000 replicates) are shown next to the branches (Felsenstein, 1985). The evolutionary distances were computed using the Maximum Composite Likelihood method (Tamura et al. 2004) and are in the units of the number of base substitutions per site. The ME tree was searched using the Close-Neighbor-Interchange (CNI) algorithm (Nei and Kumar, 2000) at a search level of 1. The Neighbor-joining algorithm (Saitou, 1987) was used to generate the initial tree. This analysis involved 30 nucleotide sequences. All ambiguous positions were removed for each sequence pair (pairwise deletion option). There were a total of 871 positions in the final dataset. Evolutionary analyses were conducted in MEGA X (Kumar et al. 2018).
